# Design and Implementation of an RTK-Based Vector Phase Locked Loop

**DOI:** 10.3390/s18030845

**Published:** 2018-03-13

**Authors:** Ahmad Shafaati, Tao Lin, Ali Broumandan, Gérard Lachapelle

**Affiliations:** Position, Location and Navigation (PLAN) Group; Schulich School of Engineering, University of Calgary, Calgary, AB T2N 1N4, Canada; shafaati.a@gmail.com (A.S.); gnsstao@outlook.com (T.L.); gerard.lachapelle@ucalgary.ca (G.L.)

**Keywords:** Global Navigation Satellite Systems (GNSS), vector tracking, carrier phase tracking, RTK

## Abstract

This paper introduces a novel double-differential vector phase-locked loop (DD-VPLL) for Global Navigation Satellite Systems (GNSS) that leverages carrier phase position solutions as well as base station measurements in the estimation of rover tracking loop parameters. The use of double differencing alleviates the need for estimating receiver clock dynamics and atmospheric delays; therefore, the navigation filter consists of the baseline dynamic states only. It is shown that using vector processing for carrier phase tracking leads to a significant enhancement in the receiver sensitivity compared to using the conventional scalar-based tracking loop (STL) and vector frequency locked loop (VFLL). The sensitivity improvement of 8 to 10 dB compared to STL, and 7 to 8 dB compared to VFLL, is obtained based on the test cases reported in the paper. Also, an increased probability of ambiguity resolution in the proposed method results in better availability for real time kinematic (RTK) applications.

## 1. Introduction

The concept of vector tracking loop (VTL) was first proposed in [1] and since then it has been of interest for Global Navigation Satellite Systems (GNSS) applications. This method leverages the spatial correlation among measurements from different satellites to enhance tracking capability. In contrast to the conventional scalar tracking loop (STL) in which each satellite signal is processed independently, VTL processes all signals in parallel using a common navigation filter. VTL is proven to have superior performance compared to STL during weak signal tracking, high dynamic situations and even under interference conditions [2,3,4,5]. VTL is based upon the fact that all received signals are spatially correlated. The receiver motion projects onto received signals from different satellites according to their corresponding locations. This property is used in vector tracking to enhance robustness. In this case, strong signals assist weak signal tracking during short time outages to prevent loss of lock. This approach is now widely used for code phase and carrier frequency tracking, leading to vector delay lock loop (VDLL) and vector frequency lock loop (VFLL) architectures, respectively. However, the methods result in a non-coherent receiver in which carrier phase measurements either are not being tracked or are tracked independently. Hence, carrier phase-based navigation algorithms such as RTK should rely on scalar-based tracking measurements.

Although vector tracking is very beneficial for frequency and code tracking loops, applying the same concept to the carrier phase is not straightforward. The reason for is that the magnitude of errors affecting GNSS signals is usually at the metre level, which is, in some cases, far more than the tolerable intervals for phase discriminators with limited linear regions. Due to relatively short GNSS carrier wavelengths, these errors can easily force the loop to drop out of lock or generate cycle slips. Hence, a significant part of disturbance in the carrier phase of each pseudo random number (PRN)is unique to the corresponding satellite, and effects such as ionospheric and tropospheric delays can make carrier phase measurement errors uncorrelated.

The Co-op tracking loop was proposed in [6] as an alternative to the vector tracking loop. Analogous to VTL, this architecture also utilizes the spatial correlations among measurements from different satellites to enhance tracking capability. In contrast, in addition to a single filter for common effects, each channel has its own channel-arm filter to track the residual phase in the corresponding channel. However, using the channel filters compromises the tracking loop performance compared to a pure vector solution, as satellite geometry is only incorporated partially [7].

Henkel et al. introduced a Multi-Frequency Multi-Carrier VPLL (MC-VPLL) in which additional information is used to keep the VPLL in lock and to account for the residual error in each channel [8]. In this case, the navigation filter also contains an ionospheric delay state for each satellite as well as a tropospheric zenith delay state. A multi-frequency receiver has been considered in order to estimate ionospheric delays. The receiver starts with scalar-based tracking loops and switches to VPLL when all tracking loops are in the locked condition and the transient regime is completed. In this way, integer ambiguities and initial phase errors are obtained from the scalar tracking loops. Results from studies using this method have shown that its outperforms scalar tracking in terms of phase jitter and the probability of loss-of-lock, provided that deep fading due to scintillation does not affect all satellites and frequencies simultaneously.

Brewer and Raquet proposed a differential VPLL (DVPLL) in which base station measurements are utilized to assist the vector loop [9]. DVPLL attempts to remove nuisance parameters, such as atmospheric errors, instead of estimating them for short to medium baselines, through differential processing of the observations. DVPLL employs single-differencing between receivers, thereby eliminating or substantially reducing atmospheric errors (for short to medium-length baselines) as well as satellite orbital and clock errors. Therefore, the receiver must estimate its own position and clock information (bias, drift, etc.). In addition, rover measurements (i.e., carrier phases and code phases) are estimated by projecting base station observations onto the rover position. In this way, the degraded incoming signals can be ignored, as the local replica is merely a function of base station observations and rover dynamics information. Hence, as long as the estimated position and clock states are fairly accurate, it is possible to generate rover observations regardless of the incoming signal quality.

In [1], a modified version of this algorithm was applied to a moving rover. It was shown that DVPLL tracking sensitivity was improved by 4 to 8 dB compared to the scalar-based tracking loop. The author conducted a moving rover experiment in a moderate to heavy foliage environment, where the vector tracking experienced a lower number of cycle slips compared to the scalar loop.

A partitioned phase tracking loop was introduced in [10] in which a combination of scalar-base and vector-based loops were employed for carrier phase tracking. The vector processing was intended to track errors caused by receiver clock bias and motion, whereas the scalar loop tracks satellite-dependent errors.

This paper introduces a double-difference VPLL (DD-VPLL) tracking architecture for carrier phase vector processing in the receiver. Similar to DVPLL, the proposed method utilizes base station observations for carrier phase estimation. However, using double-differencing obviates the necessity for estimating receivers’ clock biases. For the DVPLL case, receiver clock bias modelling with centimetre accuracy is very challenging, especially with receivers equipped with temperature-controlled oscillators (TCXO). Other effects, such as vibration-induced errors in non-stationary rovers, must be taken into account. Thus, a simple two-state clock model in DVPLL may not be adequate for dynamic cases. In order to evaluate the performance of the proposed method, a GNSS hardware simulator was employed which generates both the base station and the rover RF signals. For the rover, a circular motion was considered at constant velocity of 10 m/s with radius of 200 m.

## 2. Signal and System Model

Brewer and Raquet proposed a differential VPLL for carrier phase tracking that employs base station measurements to assist the rover tracking loops [9]. The rover carrier phase is predicted based upon base station measurements and the relative location of the two receivers. Specifically,

(1) $$\phi_{R}^{k}(t_{R})=\phi_{B}^{k}(t_{B}^{k})+f_{sat}\left(t_{B}^{k}-t_{R}\right)-\phi_{0R}^{k}+\phi_{0B}^{k}$$

where $\phi_{R}^{k}$ and $\phi_{B}^{k}$ are rover and base station carrier phase values from the *k*th satellite, $\phi_{0R}^{}$ and $\phi_{0B}^{}$ are initial phase values and $f_{sat}$ represents the GNSS carrier frequency. Accordingly, the rover carrier phase at time $t_{R}$ can be estimated by base station carrier phase at time $t_{B}^{k}$, where

(2) $$t_{B}^{k}=t_{B1}+\frac{1+\varepsilon_{B}}{1+\varepsilon_{R}}\left(t_{R}-t_{R1}\right)+\left(\frac{\left|r_{B}-r^{k}\right|-\left|r_{R}-r^{k}\right|}{c}+\Delta I^{k}+\Delta T^{k}+\Delta \eta \right)\left(1+\varepsilon_{B}\right)$$

and *c* is the speed of light, $t_{R1}$ and $t_{B1}$ are initial clock biases and $\varepsilon_{R}$ and $\varepsilon_{B}$ are clock drifts for rover and base stations. Since a base station usually has a better clock, $\varepsilon_{B}$ is smaller than 1 and $\left(1+\varepsilon_{B}\right)\approx 1$. Moreover, for short to medium baselines, the ionospheric delay ($\Delta I^{k}$) is negligible and the tropospheric delay ($\Delta T^{k}$) is insignificant, provided that the two receivers have more or less similar heights. Δη accounts for the uncorrelated errors, such as white noise and multipath, which are not modelled. $r_{B}$, $r_{R}$ and $r^{k}$ are the position vectors of base station, rover and k^th^ satellite, respectively. The authors proposed an extended Kalman filter (EKF) that estimates $t_{B1}$ and $\varepsilon_{B}$ as well as the rover’s position ($r_{R}$). Other parameters can be exploited at the rover using satellite ephemeris available at the base station. This algorithm has the advantage of eliminating common errors, such as atmospheric delays, satellites clock and orbital errors. Hence, the predicted carrier phase has a fraction of a cycle (e.g., 0.1 cycle) accuracy, provided that the rover position and clock terms can be estimated precisely.

In order to alleviate the clock bias issue, estimation and elimination are the two possible approaches. Although simplified models, such as random walk clock drift, can be utilized, they fail to model realistic clock behavior, especially for a dynamic rover with effects such as vibrations. This paper introduces the DD-VPLL tracking loop that eliminates clock bias terms and leads to a more robust loop.

Due to the double-differencing operation, measurements from a reference satellite are required. Hereafter, it is assumed that *s*_R_ refers to the reference satellite index. Based on Equation (1), it can be shown that

(3) $$\phi_{R}^{k}(t_{R})-\phi_{R}^{s_{R}}(t_{R})=\phi_{B}^{k}(t_{B}^{k})-\phi_{B}^{s_{R}}(t_{B}^{s_{R}})+f_{sat}\left(t_{B}^{k}-t_{B}^{s_{R}}\right)-\phi_{0R}^{k}+\phi_{0B}^{s_{R}}+\phi_{0R}^{k}-\phi_{0B}^{s_{R}}$$

In addition, according to Equation (2)

(4) $$t_{B}^{k}-t_{B}^{s_{R}}=\left(\frac{\left|r_{B}-r^{k}\right|-\left|r_{R}-r^{k}\right|-\left|r_{B}-r^{s_{R}}\right|+\left|r_{R}-r^{s_{R}}\right|}{c}+\Delta \nabla I^{k}+\Delta \nabla T^{k}+\Delta \nabla \eta \right)\left(1+\varepsilon_{B}\right)$$

Double differencing removes the atmospheric effects, provided that the baseline (distance between the two receivers) is relatively short (a few km), in which case the atmospheric effects are highly correlated. Hence, the carrier phase of the rover station for the k^th^ satellite can be expressed as

(5) $$\begin{array}{l} \phi_{R}^{k}\left(t_{R}\right)=\left(\phi_{B}^{k}\left(t_{B}^{k}\right)-\phi_{B}^{s_{R}}\left(t_{B}^{s_{R}}\right)+\phi_{R}^{s_{R}}\left(t_{R}\right)\right)\\ -\frac{1}{\lambda }\left(\left|r_{B}-r^{k}\right|-\left|r_{R}-r^{k}\right|-\left|r_{B}-r^{s_{R}}\right|+\left|r_{R}-r^{s_{R}}\right|+\Delta \nabla \varepsilon \right)+\Delta \nabla_{R}^{k}\phi_{0} \end{array}$$

where $\Delta \nabla_{R}^{k}\phi_{0}$ accounts for any double difference in the initial phase offset in receivers and satellites as well as integer carrier phase ambiguities. The time epoch for base station measurements can be computed as

(6) $$t_{B}^{k}=t_{R}-\frac{P_{R}^{k}-P_{B}^{k}}{c}.$$

where $P_{B}^{k}$ and $P_{R}^{k}$ are the base station and rover pseudo-range measurements for the *k*^th^ satellite. The carrier Doppler interpolation is used to estimate the base station carrier phase at any specific epoch, namely

(7) $$\phi (t_{B}^{k})=\phi ({t^{\prime }}_{B})+({t^{\prime }}_{B}-{t^{\prime }}_{B})\omega ({t^{\prime }}_{B})$$

Similarly, the carrier Doppler of the rover station can also be estimated based on the base station measurements, as

(8) $$\begin{array}{l} \omega_{R}^{k}\left(t_{B}^{k}\right)=\left(\omega_{B}^{k}\left(t_{B}^{k}\right)-\omega_{B}^{s_{R}}\left(t_{B}^{s_{R}}\right)+\omega_{R}^{s_{R}}\left(t_{R}\right)\right)-\\ \frac{1}{\lambda }\left(\left(v_{B}-v^{k}\right).e_{B}^{k}-\left(v_{R}-v^{k}\right).e_{R}^{k}-\left(v_{B}-v^{s_{R}}\right).e_{B}^{s_{R}}+\left(v_{R}-v^{s_{R}}\right).e_{R}^{s_{R}}\right) \end{array}$$

where $v_{B}$, $v_{R}$ and $v^{k}$ denote the base station, rover and the k^th^ satellite velocity vector, respectively. Also $\omega_{i}^{j}$ and $e_{i}^{j}$ are the carrier Doppler measurement and the unit directional vector between the *i*^th^ receiver and *j*^th^ satellite, respectively.

This model does not consider carrier phase multipath effects. Since multipath errors for spatially separated receivers are uncorrelated, they are not eliminated by the double differencing process. In a harsh GNSS environment with strong multipath components or in a fast fading situation, the use of the proposed DD-VPLL may not be as beneficial, as double differencing amplifies the multipath effect. Also, vector processing combines the observations from different satellites and adds correlations among them. However, if the target environment has a few strong satellites with line-of-sight (LOS) signals, e.g., partial forestry canopy, the observations that are not affected by the multipath help the navigation filter to provide a more accurate estimated receiver position and alleviate the multipath effect of other satellites. Hence, vector processing leads to better performance compared to the use of scalar loops.

Although triple differencing (TD) eliminates integer ambiguities, it is not a viable solution for carrier phase positioning. The main issue with this method is lower observability due to time differencing. In this case, the weaker geometry matrix significantly degrades the estimated position accuracy. TD is usually used to obtain a preliminary position estimate that is then used for DD RTK positioning in which case, the integer nature of the carrier phase ambiguities can be exploited; when the integer values are found, they become deterministic and are removed from the state vector, improving observability in the process.

## 3. Proposed Tracking Loop Architecture

**Figure 1:**
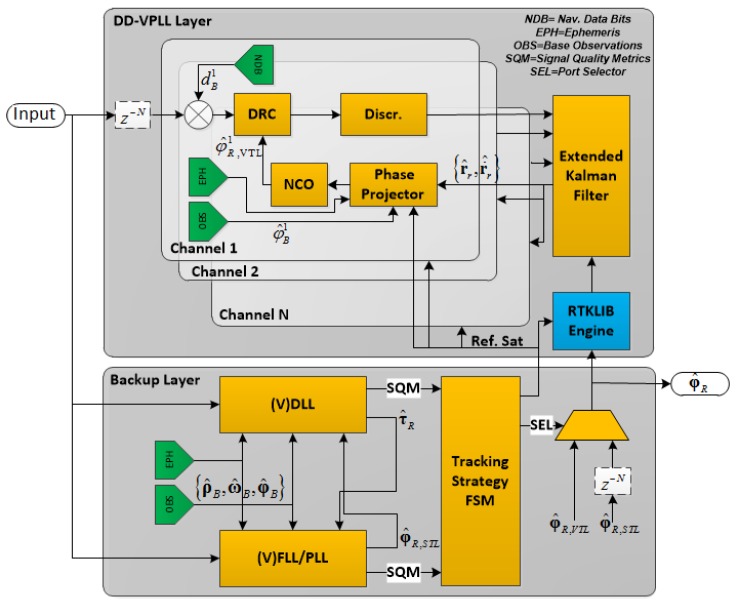
Proposed tracking loop architecture block diagram. Abbreviations: FLL, frequency locked loop; FSM, finite state machine; NCO, numerically controlled oscillator; PLL, phase-locked loop; VDLL, vector delay lock loop. (Discr. stand for discriminator and DRC stands for Doppler removal and correlation).

Figure 1 shows the overall structure of the proposed tracking loop architecture. The input signal passes through two different layers. The backup layer is a high-sensitivity tracking loop, responsible for maximizing measurement availability as well as providing reference satellite measurements. It can be a STL, VTL or any other algorithm through which signal parameters can be estimated.

The DD-VPLL layer estimates rover carrier phase values with the VPLL algorithm. The main goal of this layer is to enhance carrier phase tracking sensitivity of the receiver for situations in which the backup layer is unable to provide carrier phase measurements for a subset of satellites. An EKF is employed as the navigation filter to estimate rover position and dynamics which are then fed to phase projectors. A phase projector applies Equations (5) and (8) to estimate the rover’s carrier phase and Doppler values. Moreover, the base station aiding information is shown in green boxes in Figure 1, including navigation data bits, ephemerides and base station observations. Aiding can be utilized in the backup layer as well, to enhance its sensitivity.

Since the carrier phase prediction is very sensitive to the rover’s position error, a real time kinematic (RTK) engine is utilized in the DD-VPLL layer to initialize the navigation filter. The DD-VPLL measurements are reliable only when the RTK engine is able to fix carrier phase ambiguities. Therefore, a tracking strategy finite state machine (FSM) is added to this architecture which determines the current tracking state. Moreover, signal quality metrics (SQM), such as carrier-to-noise spectral density (C/N_0_), phase lock indicator (PLI), frequency lock indicator (FLI) and so on are fed into the algorithm to switch back and forth between DD-VPLL and STL modes.

The tracking strategy is as follows:

*Determine the tracking state*: Initially, the receiver measurements are obtained from the backup layer. As soon as the integer ambiguities are fixed, the strategy initializes the navigation filter and switches to vector mode. In this case, DD-VPLL measurements are selected as the actual observations.

*Determine the reference satellite*: Based on SQMs, the best candidate is selected to be the reference satellite. Usually, a combination of C/N_0_ and high satellite elevation angle is used for this purpose.

The EKF employed in the DD-VPLL layer estimates the rover’s position, velocity and higher order dynamics. The states vector is expressed as

(9) $$x={\left[\begin{array}{cccc} r_{R}^{T} & r_{R}^{(1)T} & \ldots & r_{R}^{(n-1)T} \end{array}\right]}_{3n\times 1}^{T}$$

where $r_{R}^{(i)T}$ is the transpose of the *i*^th^ order rover dynamic vector and *n* is the highest order of the dynamics of interest for the prediction process. Likewise, the measurement vector is defined as

(10) $$\varphi ={\left[\begin{array}{ccccccccc} \varphi^{1} & \varphi^{2} & \ldots & \varphi^{S} & \varphi^{(1),1} & \ldots & \varphi^{(1),S} & \ldots & \varphi^{(m-1),S} \end{array}\right]}_{m(S-1)\times 1}^{T}$$

where *S* is the number of satellites and *m* represents the highest order of measurement dynamics. For example, *m* = 1 when only carrier phase observations are utilized and *m* = 2 when both carrier phase and carrier–Doppler observations are used. Also, $\varphi^{(i),j}$ represents the *i*th order measurement for the *j*th satellite.

## 4. Tracking Loop Analysis

This section involves a comprehensive theoretical analysis of the proposed tracking loop. As DD-VPLL is an extension of vector tracking, it is important to determine the theoretical performance, benefits and limitations of this method compared to other tracking loop strategies.

### 4.1. Observability Analysis

Observability is an important characteristic. If the underlying model does not fulfill the observability condition, the Kalman filter as a state estimator is not applicable. This condition guarantees that the state of the system can be inferred uniquely by observing the measurements. Otherwise, given the measurements, it is not possible to determine how the states evolve, and prediction is not possible.

It is shown that a state estimator is observable if the following observability matrix is of full-rank [11]:

(11) $$O(F,H)=\left[\begin{array}{ccccc} H^{T} & F^{T}H^{T} & {\left(F^{T}\right)}^{2}H^{T} & \ldots & {\left(F^{T}\right)}^{n-1}H^{T} \end{array}\right]$$

In this section, the observability of DD-VPLL is obtained for an arbitrary number of states and measurements. According to Equation (9), the states’ dynamic matrix, **F**, can be expressed as

(12) $$F=\left[\begin{array}{ccccc} 0_{3} & I_{3} & 0_{3} & \ldots & 0_{3}\\ 0_{3} & 0_{3} & I_{3} & & 0_{3}\\ : & & & & :\\ 0_{3} & & & & I_{3}\\ 0_{3} & 0_{3} & \ldots & & 0_{3} \end{array}\right]=A_{n}⊗I_{3}$$

where ⊗ is the Kronecker product operator. $0_{n}$ and $I_{n}$ are zero and identity matrices of size *n* × *n* with

(13) $$A_{n}={\left[\begin{array}{ccccc} 0 & 1 & 0 & \ldots & 0\\ 0 & 0 & 1 & \ldots & 0\\ : & : & : & & :\\ 0 & 0 & 0 & \ldots & 1\\ 0 & 0 & 0 & \ldots & 0 \end{array}\right]}_{n\times n}$$

For the measurement model, the **H** matrix can be expressed as follows

(14) $$\begin{array}{l} H=H_{n}H_{g}=\left({\left[\begin{array}{ccccc} 1 & \frac{T}{2!} & \frac{T^{2}}{3!} & \ldots & \frac{T^{m-1}}{m!} \end{array}\right]}_{1\times m}⊗I_{S-1}\right).\left(I_{m\times n}⊗H_{g1}\right)\\ =\left[\begin{array}{ccc} 1 & \frac{T}{2} & \ldots \end{array}\right].I_{m\times n}⊗I_{S-1}.H_{g1}=H_{n1}.I_{m\times n}⊗I_{S-1}.H_{g1} \end{array}$$

where $H_{n}$ accounts for the fact that the discriminator output is the phase error average in the previous integration interval at the correlator output. $H_{g1}$ is referred to as the geometry matrix and is defined as

(15) $$H_{g1}={\left[\begin{array}{cc} e_{r}^{1} & 1\\ e_{r}^{1} & 1\\ : & :\\ e_{r}^{S} & 1 \end{array}\right]}_{S\times 4}$$

It is proven in Appendix A that the observability is guaranteed regardless of system and measurement orders, provided that at least four satellites are visible to the receiver. Hence, from A theoretical observability point of view, there is no limitation on the system and measurement order and the observability of DD-VPLL. However, the following considerations limit the system order in practice:

1. The state transition matrix (STM) of the discrete-time system is A function of the update time (*T*). In the STM equation, higher order terms are multiplied by high powers of *T*, leading to fast up/down scaling of the matrix. Hence, maintaining a high numerical precision is difficult even if numerically stable implementations are used [12].
2. Negligible third and higher order dynamics in the received GNSS signals, especially for vehicular applications [13].
3. Higher order states are weakly observable due to lack of information for estimating process noise, spectral density contents and numerical round-off errors [12].

Therefore, a system order of 2 or 3 is chosen in practice and higher order dynamics are modelled by process noise.

### 4.2. Sensitivity Analysis

When the tracking loops are locked, the discriminator outputs for all channels can be assumed to have the following Gaussian distribution vector:

(16) $$d_{\mathrm{STL}}\sim N\left(0,R_{v}\right)$$

where $R_{v}$ is the covariance matrix of the noise at the discriminator outputs. For VTL, the measurements are transferred into the position domain through the geometry matrix (**H**). The VTL can be assumed as a vector discriminator with the following distribution

(17) $$d_{\mathrm{VTL}}\sim N\left(0,H{\left(H^{T}R_{v}^{-1}H\right)}^{-1}H^{T}\right)$$

**Figure 2:**
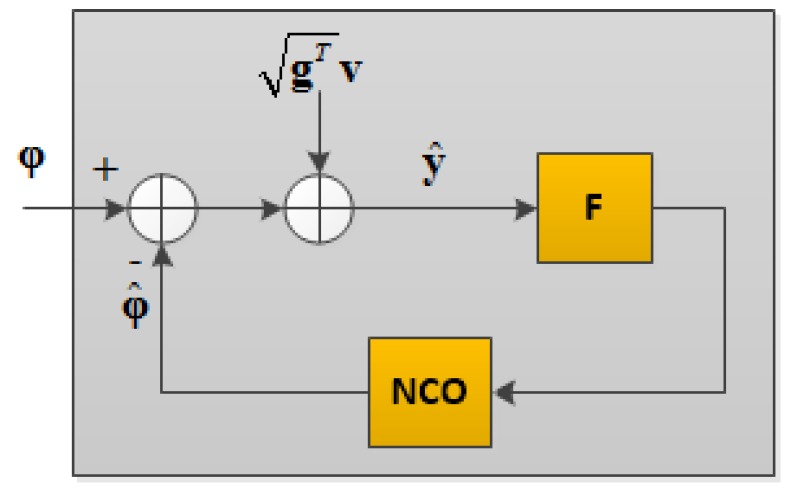
Equivalent block diagram of the vector tracking loop (VTL).

The advantage of a vector discriminator is reduction of its output noise variance by the factor

(18) $$g_{i}=\frac{\sigma_{d(\mathrm{VTL})}^{2}}{\sigma_{d(\mathrm{STL})}^{2}}=diag_{i}\left\{H.{\left(H^{T}R_{v}{}^{-1}H\right)}^{-1}.H^{T}R_{v}{}^{-1}\right\}$$

for the *i*^th^ channel. A simplified model for VTL is shown in Figure 2 in which a linearized discriminator takes the difference between the incoming carrier phase vector (φ) and the estimated vector ($\hat{\varphi }$), generated by a numerically controlled oscillator (NCO). The measurements are contaminated by the additive noise vector (v) and are passed through loop filters (F).

(19) $$g={\left[\begin{array}{cccc} g_{1} & g_{2} & \ldots & g_{n} \end{array}\right]}^{T}$$

where *n* is the number of channels that are processing in VTL and the multiplier $\sqrt{g^{T}}$ accounts for the VTL gain. In practice, when the number of satellites is more than the number of states in the navigation filter, the elements of the **g** vector are smaller than one, leading to lower tracking jitter compared to STL [3]. This shows the VTL advantages in reducing the noise of the estimated parameters.

The output variance for the coherent discriminator of an STL can be expressed as

(20) $$\sigma_{\hat{\varphi },\mathrm{STL}}^{2}=\sigma_{d(\mathrm{STL})}^{2}\int_{-\infty }^{+\infty }{\left|H_{c}(\omega )\right|}^{2}d\omega =\sigma_{d(\mathrm{STL})}^{2}.B=\frac{B}{C/N_{0}}$$

where *B* represents the equivalent noise bandwidth (ENBW), and $\sigma_{d}^{2}$ is the discriminator’s output noise variance. $H_{c}$ is a closed-loop transfer function of the loop, and $\sigma_{STL}^{2}$ denotes the tracking loop output variance. Similarly, the output vaxxriance for the *i*^th^ channel in the VTL, assuming a similar bandwidth, can be expressed as

(21) $$\sigma_{{\hat{\varphi }}_{i},\mathrm{VTL}}^{2}=\sigma_{d(\mathrm{VTL})}^{2}.B=\sigma_{d(\mathrm{STL})}^{2}.g_{i}B$$

To simplify mathematical derivations, it is assumed hereafter that all satellites have the same C/N_0_ values (i.e., $R_{v}=\sigma_{0}^{2}I_{n}$). This is the worst-case scenario because in practice it is likely that a few strong satellite observations reduce tracking jitter of the weak channels more than Equation (21).

As DD-VPLL combines the ideas of both the Difference Correlator [14] and VTL, it is expected that the performance of this tracking loop will be affected by the effects of both methods. The output tracking jitter for a Kalman-filter based DD-VPLL tracking loop can be expressed as follows ([7])

(22) $$R_{\hat{\phi },\mathrm{DDVPLL}}=\int_{-\infty }^{\infty }\left(H_{c}(\omega )R_{v}H_{c}^{H}(\omega )+\left(I-H_{c}(\omega )\right)R_{w}{\left(I-H_{c}(\omega )\right)}^{H}\right)d\omega$$

where $H_{c}$ is the closed-loop transfer of DD-VPLL, $R_{v}$ represents rover channel noise covariance and $R_{w}$ is the combined effect of the observation noise covariance at the base station and the reference satellite of the rover.

**Figure 3:**
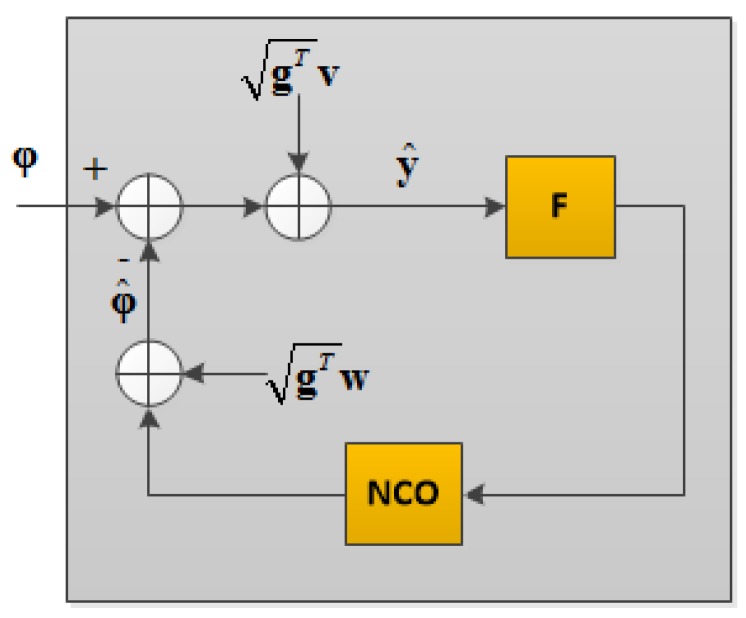
Equivalent block diagram of DD-VPLL.

In contrast to VTL, DD-VPLL adds a base station and reference satellite observations to the NCO output in order to obtain the estimated carrier phase values. Hence, the equivalent block diagram for DD-VPLL assuming vector discriminators is shown in Figure 3. Herein, the combined effects of base station and reference satellite measurements noise are referred to as **w**.

Assuming similar C/N_0_ and ENBW values for all channels in the rover and solving Equation (22), the variance of the *i*^th^ channel tracking output in the DD-VPLL can be expressed as

(23) $$\sigma_{{\hat{\varphi }}_{i},\mathrm{DDVPLL}}^{2}=g_{i}B\sigma_{v}^{2}+\left(1+g_{i}B\right)\sigma_{w}^{2}=g_{i}B\left(\sigma_{v}^{2}+\sigma_{w}^{2}\right)+\sigma_{w}^{2}$$

Compared to VTL, there are two noise sources (i.e., **v** and **w**), and the measurement noise directly affects the tracking variance. The C/N_0_ gain of DD-VPLL compared to that of STL can be expressed as

(24) $$\begin{array}{l} gain{\left(C/N_{0}\right)}_{i}=\frac{{\left(C/N_{0}\right)}_{\mathrm{STL}}}{{\left(C/N_{0}\right)}_{\mathrm{DDVPLL}}}=\frac{\sigma_{i,\mathrm{STL}}^{2}}{\sigma_{i,\mathrm{DDVPLL}}^{2}}\\ =\frac{B\sigma_{v}^{2}}{g_{i}B\left(\sigma_{v}^{2}+\sigma_{w}^{2}\right)+\sigma_{w}^{2}}=\frac{1}{g_{i}\left(1+\frac{\sigma_{w}^{2}}{\sigma_{v}^{2}}\right)+\frac{1}{B}\frac{\sigma_{w}^{2}}{\sigma_{v}^{2}}} \end{array}$$

in which the C/N_0_ gain is defined as the ratio between STL to DD-VPLL C/N_0_, resulting in the same output jitter, assuming the same loop bandwidth. Obviously, the gain is a function of the satellites’ geometry. In contrast to VTL, the DD-VPLL gain is also a function of the ratio between the tracking variance of the base station to the rover station as well as the loop bandwidth. According to the above formula, if there are no base station measurements (i.e., *w* = 0), the gain becomes similar to VTL. Moreover, the gain increases as the base station measurements have lower variances.

**Figure 4:**
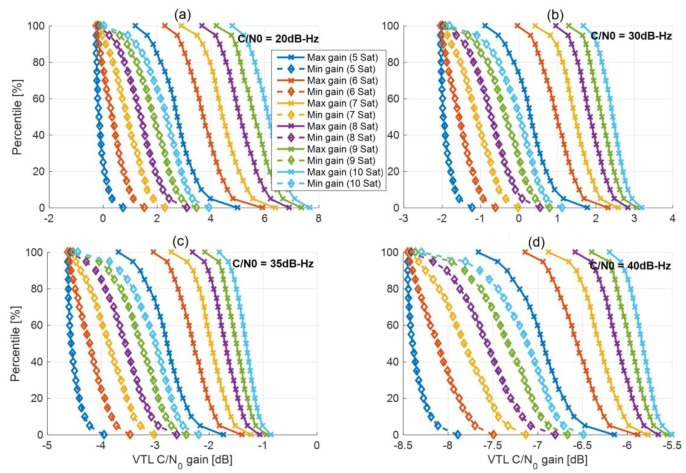
Cumulative complementary distribution function (CCDF) of DD-VPLL gain versus the number of observations for different rover carrier-to-noise spectral density (C/N_0_) values of (**a**) 20 dB-Hz; (**b**) 30 dB-Hz; (**c**) 35 dB-Hz; (**d**) 40 dB-Hz.

Figure 4 shows the cumulative complementary distribution function (CCDF) of the DD-VPLL gain for different observation numbers and rover C/N_0_ values. The simulation considers 1000 geometry samples uniformly distributed over azimuth and elevation angles (with an elevation mask of 10 degrees). The worst and the best gains are shown for each case. In this simulation, the C/N_0_ values of the base station measurements and reference satellite of the rover are assumed to be 40 dB-Hz.

In contrast to VTL, the gain can be negative, leading to a worse performance compared to STL. According to Equation (24), when the rover C/N_0_ values are 20 dB-Hz, the ratio $\frac{\sigma_{w}^{2}}{\sigma_{v}^{2}}\ll 1$. Hence, the DD-VPLL gain is almost equal to the VTL case (i.e., $\frac{1}{g_{i}}$) and is always positive (upper left subplot). Since the base station observation noise is very small in this case, the VTL gain is much larger than the loss due to the noise amplification effect of the differential operation. As the rover C/N_0_ increases, the ratio $\frac{\sigma_{w}^{2}}{\sigma_{v}^{2}}$ increases and the gain decreases (upper right and lower left subplots) due to the adverse effects of double differencing. The worst case is when the rover C/N_0_ values are equal to or greater than those of the base station (lower right subplot). In this case, the gain is always negative due to the limited VTL gain compared to the differencing operation degradation.

**Figure 5:**
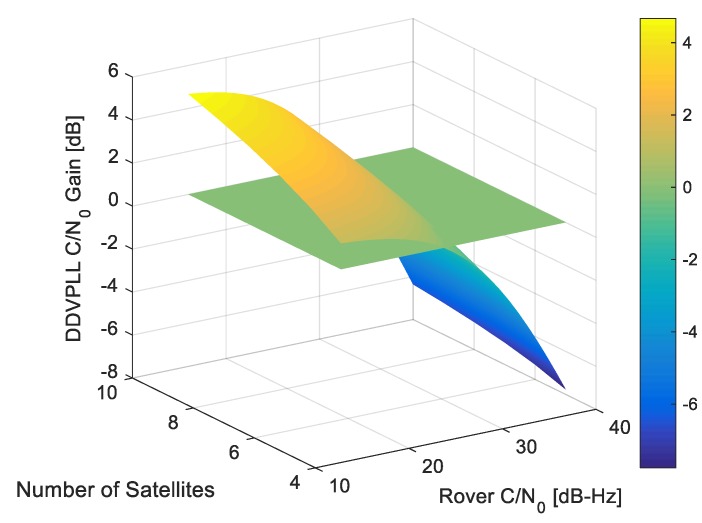
Average C/N_0_ gain of DD-VPLL for different rover C/N_0_ values and numbers of satellites.

Figure 5 shows the average C/N_0_ gain of DD-VPLL as the number of satellites and rover channels C/N_0_ values change. The 0 dB threshold is shown, to visualize the cases when DD-VPLL outperforms STL. For low C/N_0_ values, DD-VPLL has a far better performance.

## 5. Simulation Results

This section describes a simulation platform for a Monte Carlo performance evaluation of the DD-VPLL. A third order tracking loop is considered. The simulation platform generates both base station and the rover observations based on the GPS signal simulator, as described in [15]. On the receiver side, the tracking loops are implemented based on the semi-analytical simulation methodology which utilizes a correlator output model and thereby significantly reduces the number of samples to be processed [16]. Table 1 provides a list of parameters that are used for simulations in this section.

### 5.1. Performance Evaluation

**Figure 6:**
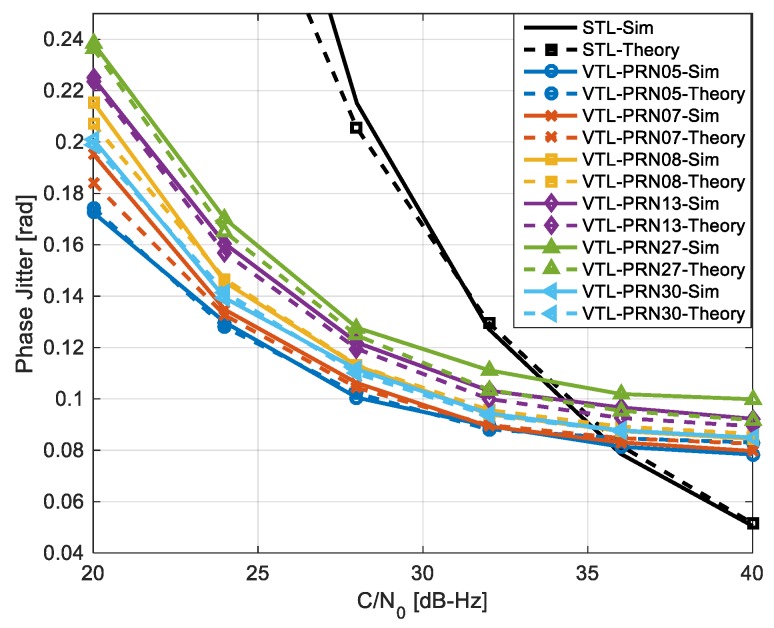
Comparing theoretical and simulation tracking phase jitter of DD-VPLL with respect to the standard tracking loops for different satellites.

This section evaluates the performance of DD-VPLL compared to STL. Herein, the worst case scenario used is defined as that when all satellite signals, including the reference, are attenuated at the same time and by the same amount. Figure 6 shows the carrier phase jitter for different C/N_0_ values. Unlike STL, the performance of different channels is not equal in the vector tracking due to its dependency on satellite geometry. For high C/N_0_ values, the tracking jitter values in DD-VPLL are slightly worse than their corresponding values in the STL. However, as the C/N_0_ decreases to 35 dB-Hz or less, the proposed method outperforms STL as the information from all satellites is used to estimate signal parameters in each channel. This simulation also verifies the theoretical analysis which resulted in Equation (24).

### 5.2. Signal Blockage Scenario

This scenario investigates the potential advantages of DD-VPLL over scalar-based tracking loops for cases where the satellites signals cannot be tracked due to partial blockages or attenuation. In these cases, strong signal observations along with known receiver/satellite positions assist the DD-VPLL to reconstruct the measurements for signals that conventional tracking cannot track. Herein, the received signals from three satellites are intentionally attenuated for 50 s (from epoch 30 s to 80 s). Initially, all satellites have similar C/N_0_ values of 40 dB-Hz. During signal blockage, three out of seven satellites (PRN 13, 27 and 28) experience severe C/N_0_ attenuation, down to 10 dB-Hz. The rover is also under a low dynamic profile with a circular motion, with a constant velocity of 10 km/h and radius of 100 m around the base station.

**Figure 7:**
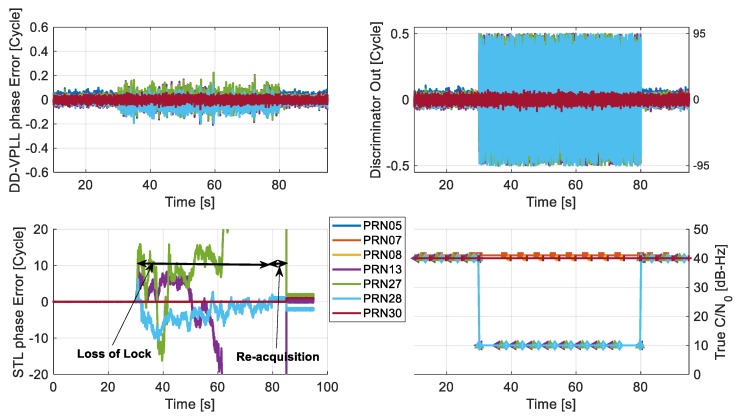
Comparison of carrier phase errors of the standard tracking loop (**c**) and DD-VPLL (**a**) with simulator reference values when the received signals of PRN 13, 27 and 28 are severely attenuated (**d**) such that their corresponding discriminator outputs have no useful information (**b**).

Figure 7 compares the tracking phase errors of DD-VPLL and STL for all satellites. Obviously, the proposed method can track attenuated PRNs properly (with tracking errors of up to 0.2 cycles) whereas the STLs fail to follow the incoming signals and experience loss of lock during the blockage interval. Also, STL requires extra re-acquisition time for recovery, with the help of the acquisition block to obtain an initial rough estimation of the signals after the attenuation interval.

## 6. Data Collection and Testing

**Figure 8:**
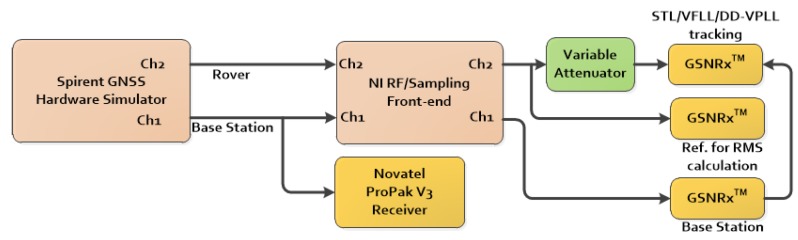
Measurement setup for data collection of hardware simulated GPS signals (NI: National Instrument, RF: Radio Frequency, Ref.: Refernece).

This section deals with actual GPS data processing and analysis. The measurement setup is shown in Figure 8. A GSS7700 Spirent hardware simulator was used to generate GPS L1/CA signals for both base and rover stations. The radio frequency (RF) signals were then down-converted, digitized and stored using a National Instrument (NI) RF front-end. Each channel used an independent internal oven-controlled crystal oscillator (OCXO) reference clock for sampling and down conversion. A NovAtel Propak-V3 receiver was utilized to generate base station observations. A stationary receiver was considered for the base station located on a building at the University of Calgary.

The GNSS software receiver, GSNRx^TM^ (University of Calgary, Calgary, Canada), developed for processing and analysis of GNSS intermediate frequency (IF) samples, was used [5]. Different variants of tracking loops were implemented in this software for processing the digitized samples. Herein, the following tracking loops implementations were considered:

- The standard scalar-based tracking loop
- The Kalman filter-based VFLL assisted PLL tracking loop [17]
- The proposed DD-VPLL method

In addition to the NovAtel receiver, a GSNRx version with standard tracking loops is utilized for processing base station samples at a high rate of 50 Hz. As shown in Figure 8, a variable attenuator was used to simulate weak signals and blockage conditions. The attenuator was implemented in the receiver after the correlators. In fact, the attenuator reduces the correlator magnitude by a constant value so that the overall C/N_0_ value decreases. Although other methods, such as the use of an external RF attenuator or hardware simulator settings to reduce the signals’ power, may seem to be more reasonable, there are two impediments which lead to a digital attenuator’s implementation:

Using a RF attenuator degrades all PRNs evenly, which is not suitable for partial signal outage situations.

One of the two available channels in the hardware simulator is used for base station observations. Hence, only one channel is available for the rover station. Also, true rover carrier phases are required for carrier phase error analysis. Hence, the rover signals are not attenuated before the receivers and a standard version of GSNRx^TM^ is utilized to generate true carrier phase values.

Carrier phase positioning and ambiguity resolution are performed by integrating open source RTKLIB software package with GSNRx^TM^. RTKLIB is comprised of a mature library that implements standard and precise positioning algorithms for GNSS [18].

### GPS Data Analysis

**Figure 9:**
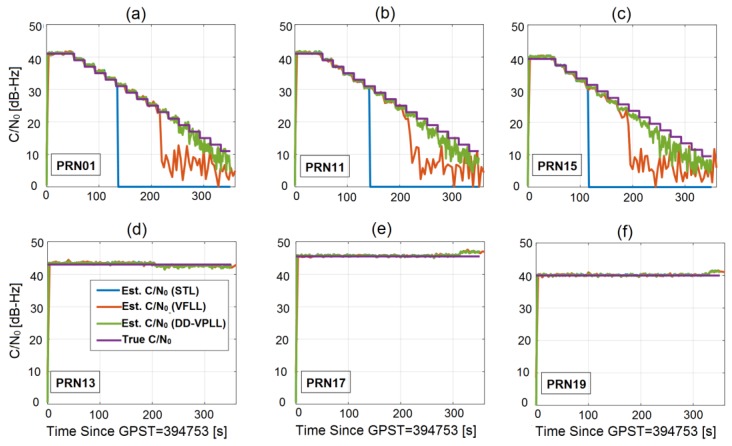
Actual and estimated C/N_0_ values for all satellites, different types of tracking loops and a circular motion rover when PRNs 01, 11 and 15 are attenuated with a staircase function over time (**a**–**c**) and the others have constant C/N_0_ values (**d**–**f**).

**Figure 10:**
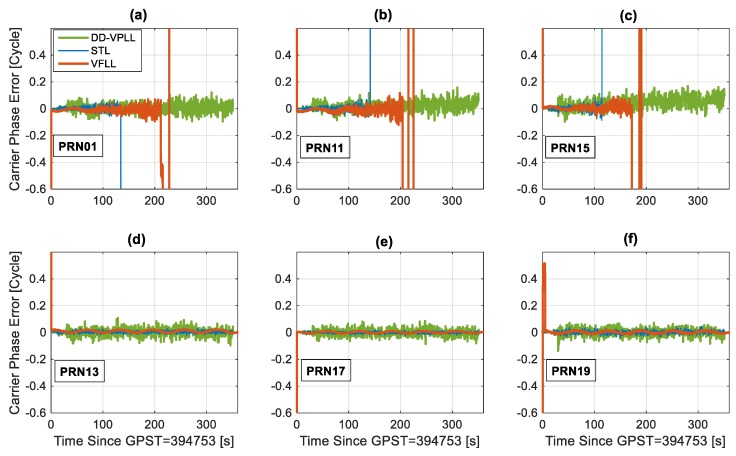
Estimated carrier phase errors for different satellites, tracking loops and a circular motion rover when PRNs of 01, 11 and 15 are attenuated with a staircase function over time (**a**–**c**) and the others have a constant C/N_0_ values (**d**–**f**).

**Figure 11:**
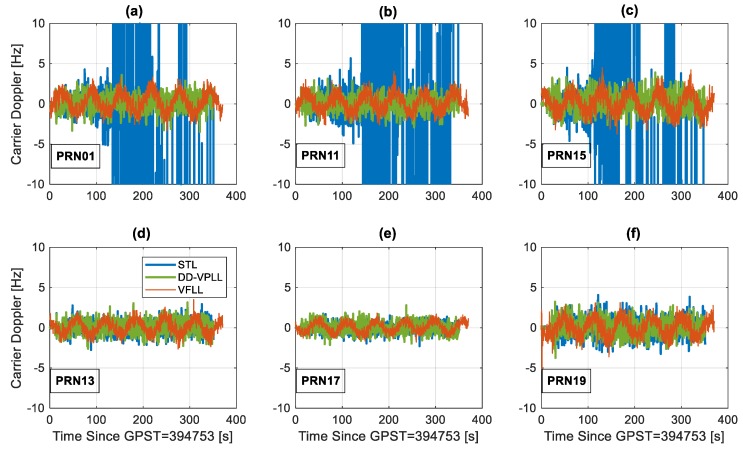
Estimated carrier Doppler errors for different satellites, tracking loops and a circular motion rover when PRNs 01, 11 and 15 are attenuated with a staircase function over time (**a**–**c**) and the others have constant C/N_0_ values (**d**–**f**).

This section compares the tracking sensitivity of different tracking loops in a typical low dynamic profile. This scenario involves a dynamic receiver with a circular motion trajectory. The base station is located at the centre of a circle with a 200-m radius. The rover has a constant velocity of 10 m/s along the circular track. It is assumed that seven satellites are in-view of the rover, and PRN28 is chosen as the reference (not shown in Figure 9, Figure 10 and Figure 11). Three satellites (PRN 1, PRN 11 and PRN 15) are attenuated at a rate of 2 dB per 20 s. The minimum observability condition of four satellites is assumed.

Figure 9 shows the true C/N_0_ values for all channels over time along with the estimated C/N_0_ values for three types of tracking loops. The estimated C/N_0_ for each channel is obtained by applying a C/N_0_ estimator on the correlator output of that channel [19]. Obviously, the estimated values are very close to the true ones when the received signals are strong. However, as the signals are more attenuated, the estimation error increases.

As shown in Figure 10, while DD-VPLL provides continuous carrier tracking, STL loses lock around 120 s and VFLL goes out of lock at 180 s for PRN 15 (the worst case). Furthermore, the estimated C/N_0_ values (i.e., Figure 9) for DD-VPLL are closer to the true values, mainly due to robust signal tracking and small tracking phase error compared to the other tracking algorithms. Nevertheless, the main drawback of the proposed tracking loop is when the signals are strong (e.g., interval 0–100 s) in which DD-VPLL has larger carrier phase errors as the differencing operation magnifies the uncorrelated noise component of the observations.

The estimated carrier Doppler errors for different satellites are shown in Figure 11. These measurements are available for a larger interval (up to 350 s for attenuated PRNs) as frequency tracking is usually less stringent than that of phase tracking. Thus, the receiver switches to the frequency lock loop (FLL) when a reliable carrier phase cannot be estimated.

**Figure 12:**
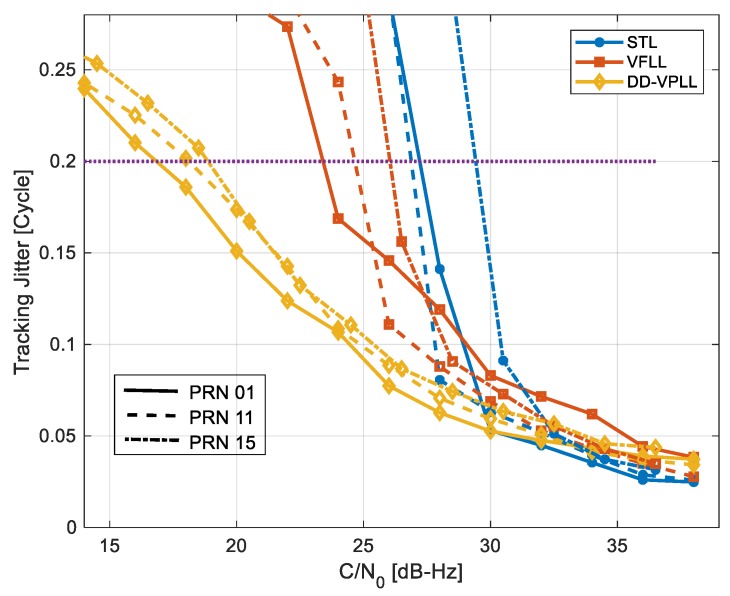
Discriminators’ output tracking jitter versus C/N_0_ values for dynamic rover scenario. The dashed line is a tracking sensitivity threshold.

Figure 12 shows the discriminator output tracking jitter values versus C/N_0_ values for different tracking algorithms. There are slight discrepancies among various satellites’ performances as the dynamic stress in the tracking loop of each channel depends on its corresponding satellite to the rover directional vector. Tracking sensitivity is defined as the minimum C/N_0_ value for which tracking jitter is below a predefined threshold. Herein, a threshold of 0.2 cycles (1/5 of the discriminator linear range) is considered for sensitivity analysis.

Table 2 provides the tracking sensitivities in this case, implying 8 to 10 dB DD-VPLL gain compared to STL, and 7 to 8 dB gain compared to VFLL.

**Figure 13:**
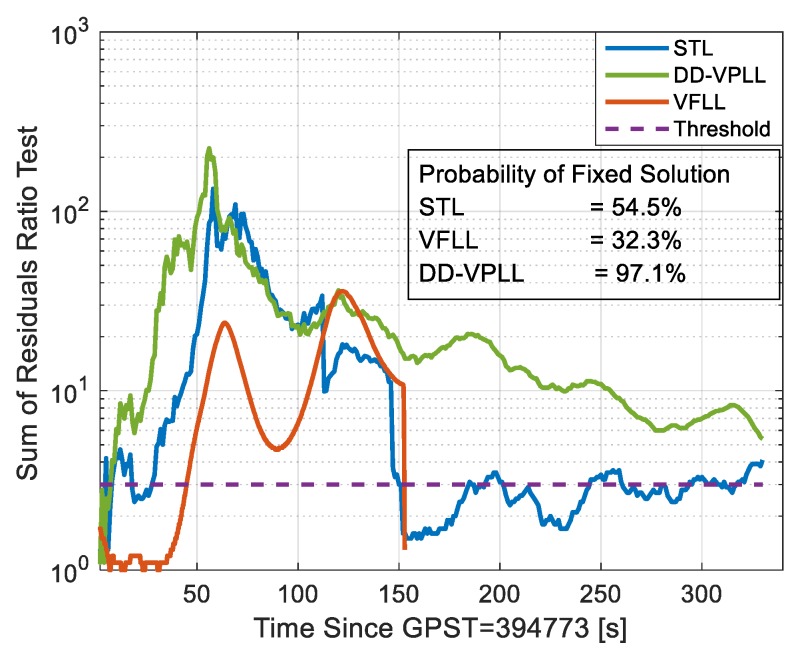
Sum of residuals ratio test results for different tracking loops.

Comparing the RTKLIB sum of residual ratio tests for different methods in Figure 13 indicates that when utilizing the proposed tracking loop, the probability of a fixed solution increases significantly. A larger ratio means that the ambiguity resolution algorithm has higher confidence in the selected integer ambiguity set [20]. When the ratio test surpasses a constant threshold, the ambiguities are used for fixed integer solution estimation. The probability of a fixed solution is defined as the time percentage that a fixed solution is available.

Herein, the RTKLIB’s ability to provide a fixed solution implies that the estimated carrier phase observations for low C/N_0_ values with the DD-VPLL approach are highly correlated with the true measurements [21].

## 7. Conclusions

In this paper a novel architecture that enhances GNSS receiver sensitivity for carrier phase tracking was introduced. The proposed DD-VPLL is a variant of vector PLL that utilizes a double differencing operation to alleviate the errors induced by the propagation channel and clock biases. The experiment, performed using sample data generated from the combination of hardware and software simulators, showed that the proposed algorithm is valid for carrier phase tracking with promising performance compared to other signal tracking methods, such as STL and VFLL. The tracking sensitivity of the receiver equipped with DD-VPLL is significantly enhanced, resulting in tracking attenuated signals down to 17 dB-Hz. A performance gain of 8 to 10 dB compared to STL and 7 to 8 dB compared to VFLL were obtained based on the test cases, indicating that this method is highly suitable in harsh signal conditions, such as partial blockages.

Comparing carrier phase positioning results, the proposed method provided a probability of fixed integer ambiguity solution of over 90% for the experiment, compared to 51% for STL and 47% for VFLL in the best case scenario.

It was shown that the tracking jitter of DD-VPLL is slightly larger than that of STL for strong signals, mainly due to noise amplification as a result of the double difference operation. However, DD-VPLL outperforms STL for medium to low C/N_0_ values (i.e., 30–35 dB-Hz) due to the vector processing gain. The threshold C/N_0_ in which DD-VPLL outperforms STL is a function of the reference satellite C/N_0_ and the number of satellites. A tracking strategy can be adopted in which observations from the backup layer are used for signals with C/N_0_ values larger than the threshold and DD-VPLL observations for other signals. In this way, the limitations of the proposed method can be reduced.

## Figures and Tables

**Table 1:** Parameters used in DD-VPLL simulations.

Simulation Parameter	Value
Reference C/N_0_	40 dB-Hz
Base station h_0_	$10^{-21}{\;\mathrm{Hz}}^{-1}$
Base station h_−2_	$10^{-24}{\;s}^{-2}{\mathrm{Hz}}^{-1}$
Rover station h_0_	$10^{-19}{\mathrm{Hz}}^{-1}$
Rover station h_−2_	$10^{-21}{\;s}^{-2}{\mathrm{Hz}}^{-1}$
Scalar tracking loops filter order	3
Scalar tracking loops filter bandwidth	15 Hz
Sampling frequency	10 MHz
Integration Time	10 ms

**Table 2:** Tracking threshold of different algorithms [dB-Hz].

Algorithm	Best Case	Worst Case
STL	26	29
VFLL	24	27
DD-VPLL	17	19

## Appendix A

According to Equations (12) and (13), it can be shown that

(A1) $$\begin{array}{ccc} {\left(F_{n}^{T}\right)}^{k}={\left(A_{n}^{T}\right)}^{k}⊗I_{3} & , & k\in \left\{1,\ldots ,n\right\} \end{array}$$

Also, for different powers of the **A** matrix, the following identities can easily be deducted:

(A2) $$\begin{array}{cccc} A_{n}^{2}=\left[\begin{array}{ccccc} 0 & 0 & 1 & \ldots & 0\\ 0 & 0 & 0 & \ldots & :\\ : & : & : & & 1\\ 0 & 0 & 0 & \ldots & 0\\ 0 & 0 & 0 & \ldots & 0 \end{array}\right]; & \ldots & A_{n}^{n-1}=\left[\begin{array}{ccccc} 0 & 0 & 0 & \ldots & 1\\ 0 & 0 & 0 & \ldots & :\\ : & : & : & & 0\\ 0 & 0 & 0 & \ldots & 0\\ 0 & 0 & 0 & \ldots & 0 \end{array}\right]; & A_{n}^{n}=0_{n} \end{array}$$

The observability matrix rank is

(A3) $$rank\left(O(F,H)\right)=rank\left(O{\left(F,H\right)}_{3n\times 3n(S-1)}\right)\leq 3n$$

Hence, the matrix is of full-rank when rank(O(F,H))=3n. Combining Equations (A1) and (14), it can be shown that

(A4) $${\left(F_{n}^{T}\right)}^{k}H^{T}=\left({\left(A_{n}^{T}\right)}^{k}⊗I_{3}\right)\left(I_{n\times m}H_{n1}^{T}⊗H_{g1}^{T}\right)={\left(A_{n}^{T}\right)}^{k}I_{n\times m}H_{n1}^{T}⊗H_{g1}^{T}$$

When a simplified measurement model is assumed with only carrier phase measurements (*m* = 1), the Equation (A4) reduces to

(A5) $${\left(F_{n}^{T}\right)}^{k}H^{T}={\left(A_{n}^{T}\right)}^{k}I_{n\times 1}⊗H_{g1}^{T}$$

Also, it can be shown that

(A6) $${\left(A_{n}^{T}\right)}^{i}{\left[\begin{array}{c} 1\\ 0\\ :\\ 0 \end{array}\right]}_{n\times 1}={\left[\begin{array}{ccccccc} 0 & \ldots & 0 & 1 & 0 & \ldots & 0 \end{array}\right]}^{T}$$

where the non-zero entry is at the *i*^th^ element of the vector. Assembling the above, the observability matrix can be written as [22]

(A7) $$\begin{array}{l} O(F,H)=\left[\begin{array}{cccc} H^{T} & F^{T}H^{T} & \ldots & {\left(F^{T}\right)}^{n-1}H^{T} \end{array}\right]\\ =\left[\begin{array}{cccc} {\left[\begin{array}{c} 1\\ 0\\ :\\ 0 \end{array}\right]}_{n\times 1}⊗H_{g1} & {\left[\begin{array}{c} 0\\ 1\\ :\\ 0 \end{array}\right]}_{n\times 1}⊗H_{g1} & \ldots & {\left[\begin{array}{c} 0\\ 0\\ :\\ 1 \end{array}\right]}_{n\times 1}⊗H_{g1} \end{array}\right]=I_{n}⊗H_{g1} \end{array}$$

It is known that rank(A⊗B)=rank(A)×rank(B). Hence

(A8) $$rank\left(O(F,H)\right)=rank\left(O{\left(F,H\right)}_{3n\times 3n(S-1)}\right)=n\times rank\left(H_{g1}\right)=n\times \min(3,S-1)$$

The above inequality is valid as long as S≥4. In other words, the system is observable as long as at least four satellites (measurements) are available.

If the measurement vector also contains higher order dynamics (i.e., *m* > 1), it is possible that some (or all) zero entries in Equation (A7) are non-zero. In this case,

(A9) $$I_{n\times m}H_{n1}^{T}=\{\begin{array}{cc} {\left[\begin{array}{cccccc} 1 & \frac{T}{2} & \ldots & \frac{T^{\left(m-1\right)}}{m!} & 0 & \begin{array}{cc} \ldots & 0 \end{array} \end{array}\right]}^{T} & m<n\\ {\left[\begin{array}{cccc} 1 & \frac{T}{2} & \ldots & \frac{T^{\left(n-1\right)}}{n!} \end{array}\right]}^{T} & m\geq n \end{array}$$

After mathematical simplifications, it can be shown that

(A10) $$O(F,H)=\left[\begin{array}{cccc} 1 & 0 & \ldots & 0\\ \frac{T}{2} & 1 & & 0\\ : & \frac{T}{2} & & :\\ \frac{T^{\left(m-1\right)}}{m!} & : & & 0\\ 0 & & & 0\\ : & 0 & & :\\ 0 & 0 & \ldots & 1 \end{array}\right]⊗H_{g1}$$

for *n* > *m* and

(A11) $$O(F,H)=\left[\begin{array}{cccc} 1 & 0 & \ldots & 0\\ \frac{T}{2} & 1 & & 0\\ : & : & & :\\ \frac{T^{\left(n-1\right)}}{n!} & \frac{T^{\left(n-2\right)}}{(n-1)!} & \ldots & 1 \end{array}\right]⊗H_{g1}$$

For m≥n, in both cases, the left-hand matrix is lower diagonal. From linear algebra, the rank of a triangular matrix is at least the number of non-zero entries on the main diagonal. Obviously, all diagonal elements in Equations (A10) and (A11) are 1 and hence, it is a full-rank matrix. In other words, the Equation (A8) is valid for any value of *m*.

## References

[1] Scott M. (2017). GPS Carrier Phase Tracking in Difficult Environments Using Vector Tracking for Precise Positioning and Vehicle Attitude Estimation Tracking in GNSS. Ph.D. Thesis.

[2] Krasovski S., Petovello M.G., Lachapelle G. Ultra-tight GPS/INS Receiver Performance in the Presence of Jamming Signals. Proceedings of the ION GNSS.

[3] Lashley M., Bevly D. (2009). What are Vector Tracking Loops, and what are their benefits and drawbacks. Inside GNSS.

[4] Pany T., Eissfeller B. Use of a Vector Delay Lock Loop Receiver for GNSS Signal Power Analysis in Bad Signal Conditions. Proceedings of the IEEE/ION PLANS.

[5] Petovello M.G., O’Driscoll C., Lachapelle G., Borio D., Murtaza H. Architecture and Benefits of an Advanced GNSS Software Receiver. Proceedings of the International Symposium on GPS/GNSS 2008.

[6] Zhodzishsky M., Yudanov S., Veitsel V., Ashjaee J. Co-Op Tracking for Carrier Phase. Proceedings of the ION GPS.

[7] Giger K.T. (2014). Multi-Signal Tracking in GNSS. Ph.D. Thesis.

[8] Henkel P., Giger K., Gunther C. (2009). Multi-Frequency, Multi-Satellite Vector Phase-Locked Loop for Robust Carrier Tracking. IEEE J. Sel. Top. Signal Process..

[9] Brewer J., Raquet J. (2016). Differential Vector Phase Locked Loop. IEEE Trans. Aerosp. Electron. Syst..

[10] Marçal J., Nunes F. Robust vector tracking for GNSS carrier phase signals. Proceedings of the 2016 International Conference on Localization and GNSS (ICL-GNSS).

[11] Gelb A., Kasper J.F., Nash R.A., Price C.F., Sutherland A.A. (2001). Applied Optimal Estimation.

[12] Brown R.G., Hwang P.Y.C. (2012). Introduction to Random Signals and Applied Kalman Filtering.

[13] Tsui J.B. (2005). Fundamentals of Global Positioning Systems Receivers. A Software Approach.

[14] Pany T., Euler H.G., Winkel J. (2012). Difference Correlators. Does Indoor Carrier Phase Tracking Allow Indoor RTK?. Inside GNSS.

[15] Dong L. (2003). IF GPS Signal Simulator Development and Verification. Master’s Thesis.

[16] Borio D., Anantharamu P.B., Lachapelle G. (2011). SATLSim: A Semi-Analytic Framework for Fast GNSS Tracking Loop Simulations. GPS Solut..

[17] Petovello M.G., Lachapelle G. Comparison of Vector—Based Software Receiver Implementations with Application to Ultra-Tight GPS/INS Integration. Proceedings of the ION GNSS.

[18] Takasu T., Yasuda A. Development of the low-cost RTK-GPS receiver with an open source program package RTKLIB. Proceedings of the International Symposium on GPS/GNSS.

[19] Van Dierendonck A.J., Parkinson B., Spilker J.J. (1993). GPS Receivers. Global Positioning System: Theory and Applications.

[20] Teunissen P.J.G. (1995). The Least-Squares Ambiguity Decorrelation Adjustment: A Method for Fast GPS Ambiguity Estimation. J. Geodesy.

[21] Lin T. (2013). Contributions to a Context-Aware High Sensitivity GNSS Software Receiver. Ph.D. Thesis.

[22] Roger A.H., Johnson C.R. (1985). Matrix Analysis.

